# *ARV1* deficiency induces lipid bilayer stress and enhances rDNA stability by activating the unfolded protein response in *Saccharomyces cerevisiae*

**DOI:** 10.1016/j.jbc.2024.107273

**Published:** 2024-04-06

**Authors:** Sujin Hong, Hyeon-geun Lee, Won-Ki Huh

**Affiliations:** 1 School of Biological Sciences, Seoul National University, Seoul, Republic of Korea; 2 Institute of Microbiology, Seoul National University, Seoul, Republic of Korea

**Keywords:** arv1, *Saccharomyces cerevisiae*, rDNA silencing, sir2, unfolded protein response (UPR), high osmolarity glycerol (HOG)

## Abstract

The stability of ribosomal DNA (rDNA) is maintained through transcriptional silencing by the NAD^+^-dependent histone deacetylase Sir2 in *Saccharomyces cerevisiae*. Alongside proteostasis, rDNA stability is a crucial factor regulating the replicative lifespan of *S. cerevisiae*. The unfolded protein response (UPR) is induced by misfolding of proteins or an imbalance of membrane lipid composition and is responsible for degrading misfolded proteins and restoring endoplasmic reticulum (ER) membrane homeostasis. Recent investigations have suggested that the UPR can extend the replicative lifespan of yeast by enhancing protein quality control mechanisms, but the relationship between the UPR and rDNA stability remains unknown. In this study, we found that the deletion of *ARV1*, which encodes an ER protein of unknown molecular function, activates the UPR by inducing lipid bilayer stress. In *arv1*Δ cells, the UPR and the cell wall integrity pathway are activated independently of each other, and the high osmolarity glycerol (HOG) pathway is activated in a manner dependent on Ire1, which mediates the UPR. Activated Hog1 translocates the stress response transcription factor Msn2 to the nucleus, where it promotes the expression of nicotinamidase Pnc1, a well-known Sir2 activator. Following Sir2 activation, rDNA silencing and rDNA stability are promoted. Furthermore, the loss of other ER proteins, such as Pmt1 or Bst1, and ER stress induced by tunicamycin or inositol depletion also enhance rDNA stability in a Hog1-dependent manner. Collectively, these findings suggest that the induction of the UPR enhances rDNA stability in *S. cerevisiae* by promoting the Msn2-Pnc1-Sir2 pathway in a Hog1-dependent manner.

The ribosomal DNA (rDNA) of *Saccharomyces cerevisiae* is composed of 100 to 200 tandemly arrayed copies of a 9.1-kb repeat on chromosome XII (1, 2). Each rDNA repeat consists of RNA polymerase (Pol) I-transcribed 35S rRNA, Pol III-transcribed 5S rRNA gene, and nontranscribed spacers (NTS1 and NTS2). The highly repetitive sequences of the rDNA locus are easy targets for homologous recombination, making the rDNA an intrinsically unstable region. Homologous recombination between rDNA repeats results in the formation of extrachromosomal rDNA circles (ERCs), and the accumulation of these ERCs to toxic levels is a major cause of replicative aging of *S. cerevisiae* (3, 4).

Under normal conditions, rDNA stability is maintained by transcriptional silencing at the rDNA locus, a process known as rDNA silencing. The regulator of nucleolar silencing and telophase exit complex is responsible for suppressing Pol II-dependent transcription at the rDNA locus and inhibiting homologous recombination between rDNA repeats (5). Sir2, an NAD^+^-dependent histone deacetylase, is a crucial subunit of the regulator of nucleolar silencing and telophase exit complex (6). Sir2 suppresses homologous recombination between rDNA repeats, leading to a decrease in the formation of ERCs and thereby extending replicative lifespan (RLS) (7).

Sir2-mediated rDNA stability is regulated by several upstream regulatory pathways, including the Msn2/4-Pnc1-Sir2 pathway (8). The enzymatic activity of Sir2 and its association with rDNA are upregulated by nicotinamidase Pnc1, which converts nicotinamide, a physiological inhibitor of Sir2, to nicotinic acid (9). *PNC1* is a well-known general stress-responsive gene regulated transcriptionally by the Msn2/4 stress-responsive transcription factors (10). It is known that Msn2/4 can be suppressed by the cAMP-PKA pathway and the target of rapamycin complex 1 (TORC1) pathway and activated by protein kinases and phosphatases, including Yak1, Snf1, Hog1, and Glc7 (11).

The endoplasmic reticulum (ER) is an organelle responsible for not only protein folding and maturation but also the synthesis and distribution of lipids (12, 13). ER stress can be induced by protein misfolding or disturbances in membrane lipid composition and can be sensed by the ER membrane protein Ire1 (14, 15). In response to ER stress, cells activate the unfolded protein response (UPR) *via* Ire1-mediated *HAC1* mRNA splicing. Hac1 stimulates the transcription of various ER stress-responsive genes, increases ER protein-folding capacity, reduces global protein synthesis, enhances autophagic degradation and ER-associated degradation of misfolded proteins, and modulates lipid metabolism (16).

The UPR exhibits intricate crosstalk with various signaling pathways, particularly the cell wall integrity (CWI) pathway (17, 18, 19). The CWI pathway, a mitogen-activated protein (MAP) kinase signaling cascade, governs CWI and cell cycle progression in response to cell wall stress (20). Notably, the CWI pathway is activated under ER stress conditions, and conversely, the UPR can be triggered under cell wall stress. Additionally, the Snf1/AMPK pathway and the high osmolarity glycerol (HOG) pathway are activated by ER stress, contributing to stress adaptation (17, 21, 22).

Recent studies have shown increased RLS in several mutants with activated UPR, suggesting a link between the UPR and yeast RLS. Notably, Ire1-dependent lifespan extension was observed in mutants such as *pmt1*Δ, *bst1*Δ, and *alg12*Δ cells (23, 24). Additionally, a lifespan extension dependent on autophagy activity, but not Ire1, was noted in *rer1*Δ cells (25). Protein oxidation and accumulation of protein aggregates, along with ERCs, are crucial factors limiting the RLS in budding yeast (4). Therefore, protein quality control and relief of ER stress through the UPR are expected to contribute to the regulation of replicative aging. However, because the RLS is determined not only by protein quality control but also by rDNA stability, an understanding of the effect of the UPR on rDNA stability is crucial for a thorough comprehension of its role in lifespan regulation.

Arv1, an ER protein, plays a crucial role in transporting glycosylphosphatidylinositol (GPI) intermediates into the ER lumen and maintaining normal intracellular sterol distribution (26, 27). These functions of Arv1 are conserved in humans, and mutations in *ARV1* in humans are known to be involved in neuronal diseases (27, 28, 29, 30). Similar to other ER proteins, the loss of Arv1 induces the UPR (31). The deletion of *ARV1* may not only impede intracellular lipid distribution but also affect the maturation of GPI-anchored proteins, potentially triggering lipid bilayer stress, proteotoxic stress, or both. However, whether Arv1 deficiency induces lipid bilayer stress or proteotoxic stress is unknown, and the interplay between activated UPR and other signaling pathways in *arv1*Δ cells remains unclear.

In the present study, we investigated the role of Arv1 dysfunction-induced UPR in regulating rDNA stability in yeast. We discovered that the deletion of *ARV1* activates the UPR, CWI, and HOG pathway. In *arv1*Δ cells, Sir2-mediated rDNA stability was increased through the activation of the Msn2-Pnc1-Sir2 pathway, and the activation of Msn2 was regulated in an Ire1-and Hog1-dependent manner. Additionally, rDNA stability was increased not only by the deletion of *ARV1* but also by pharmacologically induced ER stress. Furthermore, it was revealed that rDNA stability increases in cells deficient of other ER proteins such as Pmt1 or Bst1, suggesting that not only protein quality control but also rDNA stability may contribute to the mechanism by which the UPR regulates lifespan.

## Results

### Loss of Arv1 activates the UPR by inducing lipid bilayer stress

**Figure 1:**
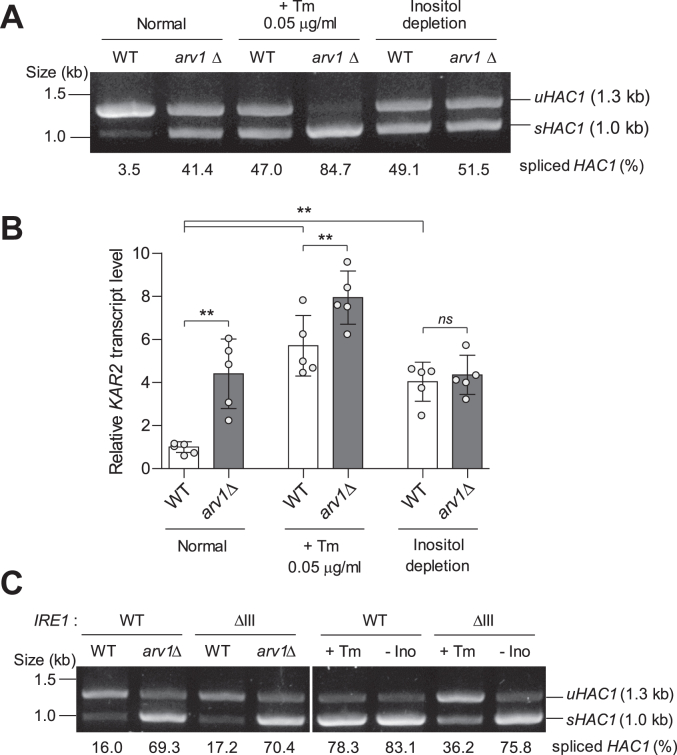
**Induction of the UPR in *arv1*Δ cells.***A*, analysis of *HAC1* mRNA splicing in WT and *arv1*Δ cells treated with or without tunicamycin (Tm) or inositol (Ino) for 3 h. *HAC1* mRNA was detected by reverse transcription-PCR. *uHAC1* and *sHAC1* indicate unspliced and spliced *HAC1*, respectively. The ratio of spliced *HAC1* to total *HAC1* is shown below each lane. Data are representative of at least three independent experiments. *B*, relative *KAR2* transcript levels in the indicated cells. Total RNA was extracted and analyzed by quantitative real-time reverse transcription-PCR. The relative *KAR2* transcript level was normalized against *TAF10* and calculated using the 2^−ΔΔCt^ method. Values represent the average of five independent experiments, and error bars indicate the standard deviation. *Asterisks* indicate significant differences (paired two-tailed Student’s *t* test): ∗∗*p* < 0.01; ns, not significant. *C*, analysis of *HAC1* mRNA splicing in cells expressing WT Ire1 or Ire1ΔIII. *HAC1* mRNA was detected by reverse transcription-PCR. *uHAC1* and *sHAC1* indicate unspliced and spliced *HAC1*, respectively. The ratio of spliced *HAC1* to total *HAC1* is shown below each lane. Data are representative of at least three independent experiments. UPR, unfolded protein response.

Induction of the UPR pathway leads to the excision of a 252-nucleotide intron from the mRNA of *HAC1*, which encodes a basic leucine zipper transcription factor responsible for regulating the UPR (32). To verify whether the deletion of *ARV1* induces the UPR, we examined *HAC1* mRNA splicing. In WT cells, the majority of the detected *HAC1* mRNA remained unspliced, indicating low basal UPR activity (Fig. 1*A*). Consistent with a previous report (31), *ARV1* deficiency resulted in elevated basal *HAC1* mRNA splicing. Treatment with tunicamycin, which induces extensive protein misfolding (33), and depletion of inositol, which induces lipid bilayer stress (34), markedly increased the fraction of spliced *HAC1* mRNA. Additionally, we assessed the extent of UPR induction by analyzing the transcript level of *KAR2*, a gene encoding an ER chaperone that is upregulated by UPR activation (35, 36). We observed increased *KAR2* transcript levels upon the deletion of *ARV1*, tunicamycin treatment, and inositol depletion (Fig. 1*B*).

Upon tunicamycin treatment, the absence of Arv1 further increased *HAC1* mRNA splicing and *KAR2* transcription (Fig. 1, *A* and *B*). However, under inositol-depleted conditions, the deficiency of Arv1 did not affect UPR activation. These findings lead us to infer that the loss of Arv1 induces the same type of ER stress as observed in inositol depletion. To validate this hypothesis, we constructed an Ire1 mutant (*IRE1*ΔIII) with the deletion of core region III in the ER luminal domain (34, 37, 38). This mutant is unable to bind misfolded proteins and thus cannot mediate the UPR triggered by proteotoxic stress, while it can normally sense ER stress induced by lipid bilayer stress (34). In contrast to Ire1, Ire1ΔIII did not sufficiently splice *HAC1* mRNA upon tunicamycin treatment (Fig. 1*C*). Conversely, in conditions of inositol depletion and Arv1 deficiency, Ire1ΔIII spliced *HAC1* mRNA similarly to Ire1. Taken together, these results suggest that the loss of Arv1 induces lipid bilayer stress and activates the UPR, similar to inositol-depleted conditions.

### Loss of Arv1 activates the MAP kinase Hog1 in an UPR-dependent manner

**Figure 2:**
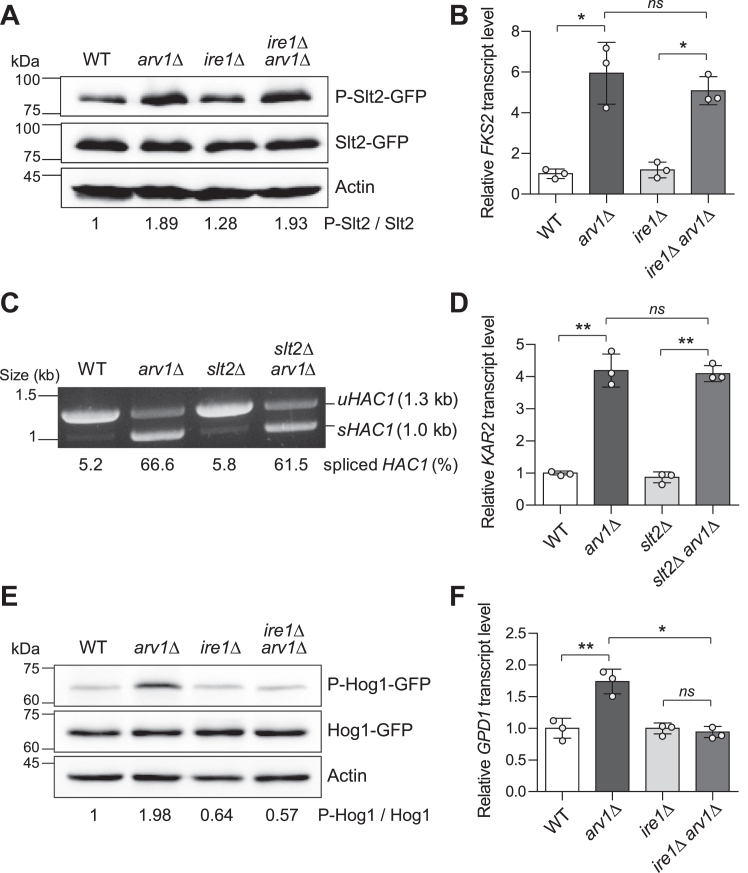
**Ire1-dependent Hog1 activation in *arv1*Δ cells.***A*, phosphorylation and total protein levels of Slt2 in WT, *arv1*Δ, *ire1*Δ, and *arv1*Δ *ire1*Δ cells. *B*, relative *FKS2* transcript levels in the indicated cells. *C*, analysis of *HAC1* mRNA splicing in WT, *arv1*Δ, *slt2*Δ, and *arv1*Δ *slt2*Δ cells. *HAC1* mRNA was detected by reverse transcription-PCR. *uHAC1* and *sHAC1* indicate unspliced and spliced *HAC1*, respectively. The ratio of spliced *HAC1* to total *HAC1* is shown below each lane. Data are representative of at least three independent experiments. *D*, relative *KAR2* transcript levels in the indicated cells. *E*, phosphorylation and total protein levels of Hog1 in WT, *arv1*Δ, *ire1*Δ, and *arv1*Δ *ire1*Δ cells. *F*, relative *GPD1* transcript levels in the indicated cells. For *A* and *E*, total protein was extracted and immunoblotting was performed using an anti-phospho-p44/42 MAPK (Erk1/2) antibody and an anti-phospho-p38 antibody for the detection of phosphorylated Slt2 and Hog1, respectively. A mouse anti-GFP antibody for the detection of GFP-tagged Slt2 or Hog1. Act1 was used as a loading control. The relative ratio of phosphorylated protein to total protein, normalized against that of WT cells, is shown below each lane. Data are representative of at least three independent experiments. For *B*, *D* and *F*, total RNA was extracted and analyzed by quantitative real-time reverse transcription-PCR. The relative transcript level was normalized against *TAF10* and calculated using the 2^−ΔΔCt^ method. Values represent the average of three independent experiments, and error bars indicate the standard deviation. *Asterisks* indicate significant differences (paired two-tailed Student’s *t* test): ∗*p* < 0.05; ∗∗*p* < 0.01; ns, not significant. HOG, high osmolarity glycerol; MAP, mitogen-activated protein.

Cellular processes, such as protein synthesis, modification, and lipid synthesis in the ER, critically influence the functions of various cellular organelles, particularly contributing to the maintenance of CWI (39). The CWI pathway can be activated under ER stress conditions such as tunicamycin treatment or DTT treatment, and cell wall stress conditions such as calcofluor white treatment or Congo red treatment can also activate the UPR (17, 18). Slt2, a MAP kinase of the CWI pathway, becomes phosphorylated upon stimulation of the CWI pathway (20). This phosphorylation activates Slt2, leading to the expression of CWI target genes, including *FKS2*, which encodes a catalytic subunit of 1,3-β-glucan synthase (20, 40). To investigate whether the deletion of *ARV1* activates the CWI pathway, we examined the phosphorylation levels of Slt2 by immunoblotting. As shown in Figure 2*A*, the loss of Arv1 increased the phosphorylation of Slt2. The transcript level of *FKS2* was also elevated in *arv1*Δ cells (Fig. 2*B*). Notably, Ire1 was not required for Slt2 activation induced by Arv1 deficiency. Next, we investigated whether Slt2 is involved in the activation of the UPR in *arv1*Δ cells. The absence of Slt2 did not affect basal UPR activity (Fig. 2, *C* and *D*). In *arv1*Δ *slt2*Δ cells, both *HAC1* mRNA splicing and the transcript level of *KAR2* were similar to those observed in *arv1*Δ cells. These results suggest that the loss of Arv1 activates the UPR and the CWI pathway independently of each other.

ER stress can activate diverse signaling pathways beyond the CWI pathway. Previous studies have reported that the Snf1/AMPK pathway and the HOG pathway can be activated by ER stress (17, 21, 22). The Snf1/AMPK pathway mediates the cellular response to nutrient availability, particularly glucose limitation, by modulating the transcription of genes involved in carbon utilization and respiration (41). Under conditions of glucose limitation, Snf1, a catalytic subunit of the AMPK complex in *S. cerevisiae*, is activated by phosphorylation (42). To explore whether the deletion of *ARV1* activates the Snf1/AMPK pathway, we examined the phosphorylation levels of Snf1 by immunoblotting. Unlike Slt2, it was observed that the loss of Arv1 did not increase the phosphorylation of Snf1 (data not shown).

Hog1 is a MAP kinase of the HOG pathway. Hog1 is activated by phosphorylation and stimulates the expression of stress-responsive genes under various stress conditions, including osmostress and ER stress (43, 44). To investigate whether the deletion of *ARV1* activates Hog1 and whether the activation of Hog1 is associated with the UPR, we examined the phosphorylation levels of Hog1 by immunoblotting. The loss of Arv1 increased the phosphorylation of Hog1 without altering the expression levels of Hog1 (Fig. 2*E*). Notably, in the absence of Ire1, which mediates the UPR by regulating Hac1 synthesis through *HAC1* mRNA splicing, the loss of Arv1 did not elevate Hog1 phosphorylation. This result suggests that the loss of Arv1 activates Hog1 in a UPR-dependent manner. *GPD1* encodes a glycerol-3-phosphate dehydrogenase, a key enzyme involved in glycerol synthesis, and is well known as a Hog1–dependent stress response gene (45, 46). To validate the above result that *ARV1* deletion activates Hog1, we measured the expression levels of *GPD1* using real-time reverse transcription PCR analysis. As shown in Figure 2*F*, the transcript levels of *GPD1* were significantly increased in *arv1*Δ cells. Furthermore, the upregulation of *GPD1* induced by *ARV1* deficiency was abolished upon the deletion of *IRE1*. This result reinforces the notion that the activation of Hog1 in the absence of Arv1 is dependent on Ire1. Taken together, these results suggest that the loss of Arv1 activates two MAP kinases, Slt2 and Hog1, and that Ire1 is involved in the activation of Hog1 in Arv1-deficient cells.

### Loss of Arv1 promotes rDNA stability in Ire1-and Hog1-dependent manner

**Figure 3:**
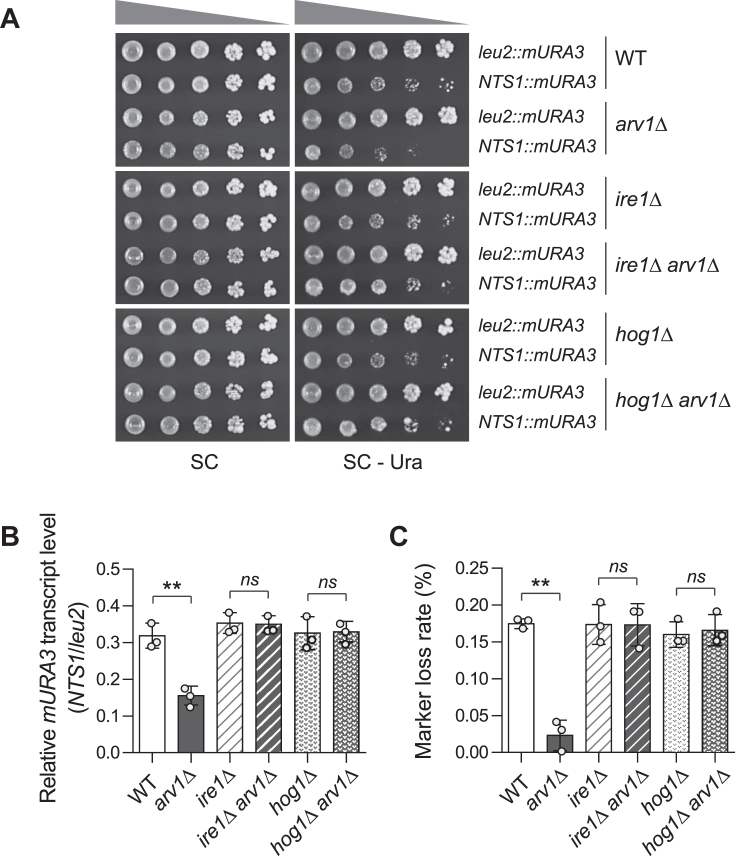
**Ire1-and Hog1-dependent enhancement of rDNA stability in *arv1*Δ cells.***A*, rDNA silencing assay performed with WT, *arv1*Δ, *ire1*Δ, *arv1*Δ *ire1*Δ, *hog1*Δ, and *arv1*Δ *hog1*Δ cells. Silencing at the rDNA region was assessed by monitoring the growth of 10-fold serial dilutions of cells on SC media lacking uracil. SC medium was used as a plating control. *B*, relative *mURA3* transcript levels in the indicated cells. Total RNA was extracted and analyzed by quantitative real-time reverse transcription-PCR. The relative transcript levels of the *mURA3* gene were calculated as the ratio of the normalized transcript levels of the *mURA3* gene inside the rDNA array (*NTS1::mURA3*) to those outside the rDNA array (*leu2::mURA3*). *C*, rDNA recombination assay performed with the indicated cells. rDNA recombination is represented by the frequency of loss of the *ADE2* marker gene integrated at the rDNA locus in the corresponding cells. For *B* and *C*, values represent the average of three independent experiments, and error bars indicate the standard deviation. *Asterisks* indicate significant differences (paired two-tailed Student’s *t* test): ∗∗*p* < 0.01; ns, not significant. HOG, high osmolarity glycerol; NTS, nontranscribed spacer; rDNA, ribosomal DNA; SC, synthetic complete.

Our previous studies revealed that the deletion of *GAS1* and *SMI1*, which are involved in the CWI pathway, increases rDNA stability in a Slt2-and Hog1-dependent manner, respectively (47, 48). We wondered whether rDNA stability is also increased by the deletion of *ARV1* and whether Slt2 and Hog1 are involved in this process. To examine whether the loss of Arv1 enhances Sir2-mediated rDNA silencing, we used yeast strains harboring the *mURA3* silencing reporter gene integrated either inside the rDNA locus (*RDN1-NTS1::mURA3*) or outside the rDNA locus (*leu2::mURA3*) (49) and monitored transcriptional silencing of *mURA3* at the rDNA locus using spot assay and quantitative real-time reverse transcription PCR analysis. In WT cells, the *mURA3* reporter gene was efficiently silenced at the rDNA locus (Fig. 3, *A* and *B*). Compared with WT cells, *arv1*Δ cells showed increased *mURA3* silencing at the rDNA locus. Reexpression of the *ARV1* gene in *arv1*Δ cells restored the rDNA silencing of *arv1*Δ cells to a level similar to that of WT cells (Fig. S1, *A* and *B*). Moreover, *arv1*Δ *sir2*Δ cells exhibited impaired rDNA silencing similar to that of *sir2*Δ cells, indicating that the loss of Arv1 increases Sir2-mediated rDNA silencing. *arv1*Δ *slt2*Δ cells showed rDNA silencing similar to that of *arv1*Δ cells, suggesting that rDNA silencing in *arv1*Δ cells is regulated independently of Slt2. Notably, enhanced transcriptional silencing of *mURA3* at the rDNA locus in *arv1*Δ cells was abolished by the deletion of *IRE1* or *HOG1* (Fig. 3, *A* and *B*). These results suggest that Ire1 and Hog1 are required for Sir2-mediated rDNA silencing increased by Arv1 deficiency.

We next examined rDNA stability by measuring the rDNA recombination rate, assessing the frequency of loss of the *ADE2* marker gene integrated at the rDNA locus (7). Consistent with the above observation of increased rDNA silencing in *arv1*Δ cells, Arv1 deficiency reduced the rate of *ADE2* marker loss (Fig. 3*C*). This phenotype was restored by reexpression of *ARV1* in *arv1*Δ cells and abolished by the deletion of *SIR2* (Fig. S1*C*). *arv1*Δ *slt2*Δ Cells exhibited decreased rDNA recombination similar to that of *arv1*Δ cells. Consistent with the above results, the deletion of *IRE1* or *HOG1* abolished enhanced rDNA stability in *arv1*Δ cells (Fig. 3*C*). Taken together, these results suggest that Arv1 deficiency increases rDNA stability by enhancing Sir2-mediated rDNA silencing in Ire1-and Hog1-dependent manners, and Slt2 is not involved in this process.

### Loss of Arv1 promotes the Msn2/4-Pnc1-Sir2 axis by activating Hog1

**Figure 4:**
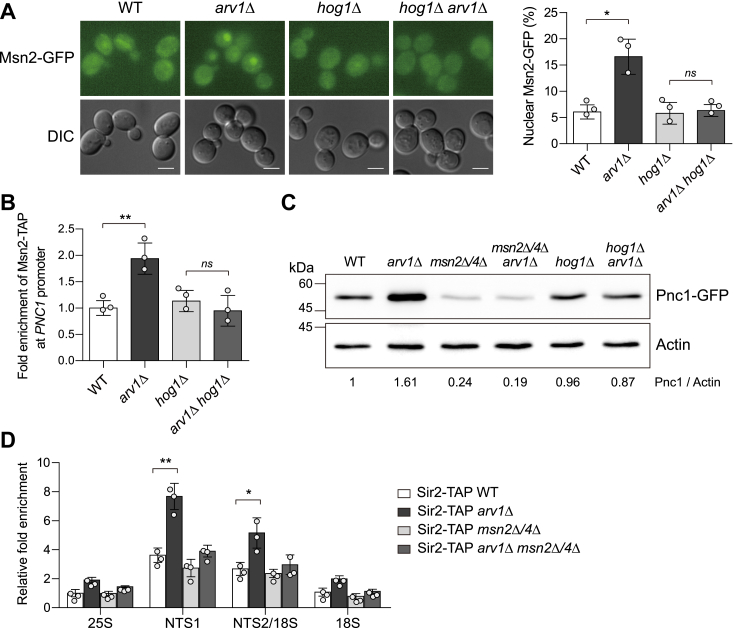
**Hog1-dependent nuclear accumulation of Msn2 and expression of Pnc1, leading to the enhancement of Sir2 association with rDNA region in *arv1*Δ cells.***A*, subcellular localization of Msn2 in WT, *arv1*Δ, *hog1*Δ, and *arv1*Δ *hog1*Δ cells. Exponentially growing cells with chromosomally GFP-tagged Msn2 in SC medium was analyzed by fluorescence microscopy (*left panel*). The scale bars represent 2 μm. The percentage of nuclear Msn2 was calculated (*right panel*). At least 200 cells were counted for each determination. *B*, association of Msn2 at the *PNC1* promoter measured using a ChIP assay in WT, *arv1*Δ, *hog1*Δ, and *arv1*Δ *hog1*Δ cells. *C*, Pnc1 protein levels in WT, *arv1*Δ, *msn2*Δ/*4*Δ, *arv1*Δ *msn2*Δ/*4*Δ, *hog1*Δ, and *arv1*Δ *hog1*Δ cells. Total protein was extracted, and immunoblotting was performed using a mouse anti-GFP antibody for the detection of GFP-tagged Pnc1. Act1 was used as a loading control. The relative ratio of Pnc1 to Act1, normalized against that of WT cells, is shown below each lane. Data are representative of at least three independent experiments. *D*, association of Sir2 at the rDNA region measured using a ChIP assay in WT, *arv1*Δ, *msn2*Δ/*4*Δ, and *arv1*Δ *msn2*Δ/*4*Δ cells. The association of Sir2 with four representative regions in the rDNA locus (25S, NTS1, NTS2/18S, and 18S regions) was analyzed by quantitative real-time reverse transcription-PCR. Values represent the average of three independent experiments, and error bars indicate the standard deviation. *Asterisks* indicate significant differences (paired two-tailed Student’s *t* test): ∗*p* < 0.05; ∗∗*p* < 0.01; ns, not significant. ChIP, chromatin immunoprecipitation; HOG, high osmolarity glycerol; NTS, nontranscribed spacer; rDNA, ribosomal DNA; SC, synthetic complete.

In *S. cerevisiae*, the transcription factors Msn2 and Msn4 regulate the general stress response. Under various stress conditions, Msn2/4 translocate from the cytosol to the nucleus, stimulating the expression of stress-responsive genes by binding to the stress response elements located in the promoters of these genes (50). One of these genes, *PNC1*, encodes nicotinamidase, and increased expression of Pnc1 enhances Sir2 activity, thereby promoting rDNA stability (8). In our previous study, we revealed that the activation of Hog1 promotes the Msn2/4-Pnc1-Sir2 pathway in the absence of Smi1, a protein involved in cell wall synthesis and maintenance (48). To examine whether Hog1 activation in *arv1*Δ cells also promotes the Msn2/4-Pnc1-Sir2 pathway, we investigated the localization of Msn2 in *arv1*Δ cells. Remarkably, the loss of Arv1 induced the nuclear accumulation of Msn2 (Fig. 4*A*). The nuclear accumulation of Msn2 in *arv1*Δ cells was abolished in the absence of Hog1, suggesting that the effect of Arv1 on the nuclear accumulation of Msn2 is dependent on Hog1.

Next, we wondered whether Msn2 accumulated in the nucleus increases the expression of Pnc1 in *arv1*Δ cells. To investigate whether nuclear-localized Msn2 binds to the *PNC1* promoter, we measured the association of Msn2 with the *PNC1* promoter using a chromatin immunoprecipitation (ChIP) assay in yeast strains in which the endogenous *MSN2* gene was modified to produce a C-terminal tandem affinity purification fusion protein. Remarkably, the loss of Arv1 increased the binding of Msn2 to the *PNC1* promoter in a Hog1-dependent manner (Fig. 4*B*). We also observed that the expression levels of Pnc1 were increased in *arv1*Δ cells (Fig. 4*C*). In the absence of Msn2/4, however, the loss of Arv1 did not increase the expression levels of Pnc1. Increased expression levels of Pnc1 in *arv1*Δ cells were also diminished in the absence of Hog1. These observations suggest that Arv1 deficiency results in an increase in Pnc1 levels through the binding of Msn2/4 to the *PNC1* promoter, which is induced by the activation of the HOG pathway.

Elevated expression of Pnc1 not only increases the catalytic activity of Sir2 but also enhances the binding of Sir2 to NTS regions in the rDNA locus (51). To examine whether elevated Pnc1 levels enhance the association of Sir2 with the rDNA locus in *arv1*Δ cells, we constructed yeast strains in which the endogenous *SIR2* gene was modified to produce a C-terminal tandem affinity purification fusion protein. A ChIP assay was conducted for four representative regions of the rDNA locus—the 25S, NTS1, NTS2/18S, and 18S regions. Consistent with the results described above, *arv1*Δ cells showed further enrichment of Sir2 at the NTS1 and NTS2/18S regions, and the enhanced association of Sir2 with the rDNA locus in *arv1*Δ cells was abolished by the deletion of *MSN2*/*4* (Fig. 4*D*). Taken together, these results suggest that Arv1 deficiency leads to an increased association of Sir2 with the rDNA locus by upregulating the expression of Pnc1, which is driven by the nuclear accumulation of Msn2/4 in a Hog1-dependent manner.

### rDNA stability is enhanced during general ER stress

Given that the loss of Arv1 activates the UPR and the subsequent activation of Hog1 increases rDNA stability, we wondered whether rDNA silencing and stability would also be enhanced under other ER stress conditions. To test this, we examined rDNA silencing and stability when proteotoxic stress and lipid bilayer stress were induced by tunicamycin treatment and inositol depletion, respectively. Both rDNA silencing and stability increased in WT cells treated with 0.05 μg/ml tunicamycin (Figs. S2, *A* and *B*, and 5*A*). Additionally, rDNA silencing and stability were increased in WT cells in inositol-depleted medium. In contrast, the increase in rDNA silencing and stability due to inositol depletion or tunicamycin treatment was not observed in *hog1*Δ cells. These results suggest that proteotoxic stress and lipid bilayer stress enhance rDNA silencing and stability in a Hog1-dependent manner. Correlating with these results, the loss of Ost3, which is involved in protein N-glycosylation and induces proteotoxic stress (38), and Opi3, which is involved in phosphatidylcholine synthesis and induces lipid bilayer stress (52), increased rDNA stability (Fig. S3, *A* and *B*).

**Figure 5:**
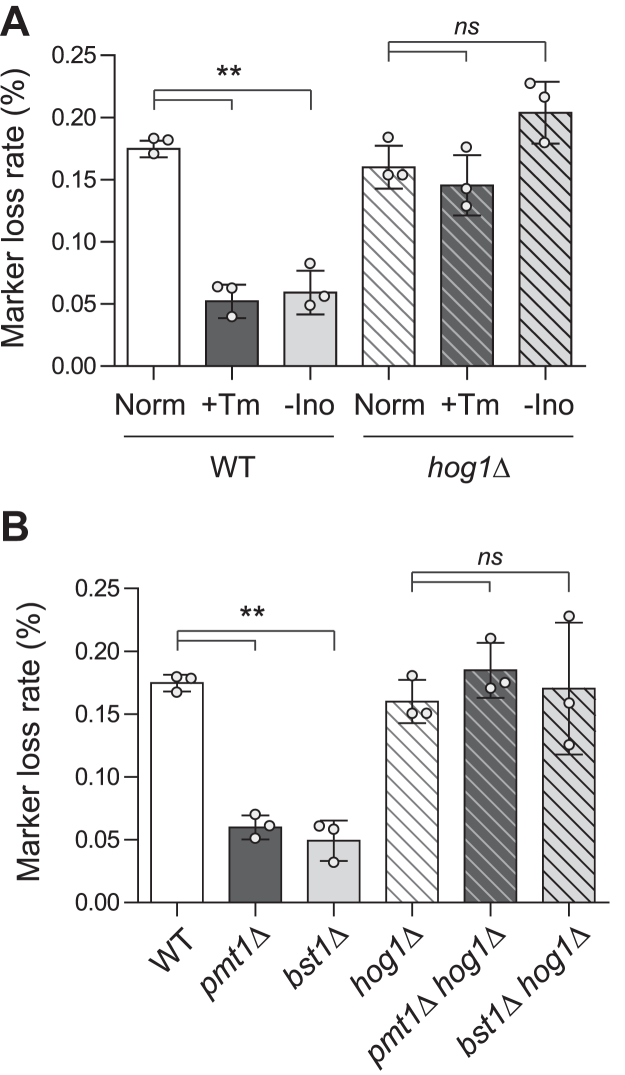
**Enhancement of rDNA stability by various ER stress conditions.***A*, rDNA recombination assay performed with WT and *hog1*Δ cells the indicated cells under 0.05 μg/ml Tm treatment or inositol depletion. *B*, rDNA recombination assay performed with WT, *pmt1*Δ, *bst1*Δ, *hog1*Δ, *pmt1*Δ *hog1*Δ, and *bst1*Δ *hog1*Δ cells. rDNA recombination is represented by the frequency of loss of the *ADE2* marker gene integrated at the rDNA locus in the corresponding cells. Values represent the average of three independent experiments, and error bars indicate the standard deviation. *Asterisks* indicate significant differences (paired two-tailed Student’s *t* test): ∗∗*p* < 0.01; ns, not significant. rDNA, ribosomal DNA.

Dysfunction of various ER secretory pathway-related proteins can also induce UPR activation. Among them, it has been reported that the deletion of *PMT1* or *BST1* not only activates the UPR but also extends the RLS (23, 24). Considering that rDNA stability is a crucial factor for maintaining the RLS, an increase in rDNA stability can be expected in *pmt1*Δ and *bst1*Δ cells. To verify this, we investigated rDNA silencing and stability in these mutants. As shown in Fig. S2, *C* and *D*, both *pmt1*Δ and *bst1*Δ cells exhibited enhanced rDNA silencing, and this increase in rDNA silencing was abolished by the deletion of *HOG1*. Furthermore, the deletion of *PMT1* and *BST1* also promoted rDNA stability (Fig. 5*B*). Similar to the deletion of *ARV1*, the increase in rDNA stability resulting from the deletion of *PMT1* and *BST1* was regulated in a Hog1-dependent manner. Considering that both Arv1 and Bst1 are involved in GPI anchor synthesis (53, 54), it is presumable that rDNA stability may also increase in other mutants that inhibit GPI anchor synthesis. Indeed, the deletion of *GPI1*, which encodes a GPI-N-acetylglucosaminyltransferase involved in the first step of GPI anchor synthesis (55), also increased rDNA stability (Fig. S3*C*). Taken together, these results suggest that both proteotoxic stress and lipid bilayer stress, as well as defects in GPI anchor synthesis, can increase rDNA silencing and stability in a Hog1-dependent manner. Additionally, UPR activation appears to contribute positively to yeast lifespan by enhancing rDNA stability.

## Discussion

Arv1 is a cortical ER protein required for normal intracellular sterol distribution, and recent studies have proposed its involvement in the synthesis of GPI anchors (27, 31, 54). Therefore, the deletion of *ARV1* disrupts the normal distribution of sterols, inhibits the maturation of GPI-anchored proteins, causes proteins to accumulate in the ER, and is likely to induce both lipid bilayer stress and proteotoxic stress. We confirmed that the loss of Arv1 primarily induces lipid bilayer stress using a mutant form of Ire1 (Fig. 1*C*). The IreΔIII mutant is widely used to distinguish between proteotoxic stress and lipid bilayer stress due to its inability to detect misfolded proteins (34, 37, 38). A recent study suggested that, while the luminal domain of Ire1 is required to detect proteotoxic stress, some residues in the amphipathic and transmembrane helices of Ire1 may be required to detect lipid bilayer stress (37, 38). Furthermore, it is essential to consider whether the lipid bilayer stress resulting from Arv1 deficiency primarily stems from defects in sterol metabolism or inhibition of GPI anchor synthesis. Previous studies have shown that the inhibition of GPI anchor synthesis induced by Arv1 can be mitigated at high temperatures (54). However, we found that the UPR activated in *arv1*Δ cells persisted even at elevated temperatures (data not shown). Further investigation is warranted to elucidate the detailed mechanism of UPR caused by Arv1 deficiency. Meanwhile, we also investigated the effect of Arv1 overexpression on the UPR. In contrast to the deletion of *ARV1*, the overexpression of Arv1 did not affect *HAC1* mRNA splicing nor *KAR2* transcription (Fig. S4). This observation suggests that *ARV1* affects the UPR only upon its deletion.

**Figure 6:**
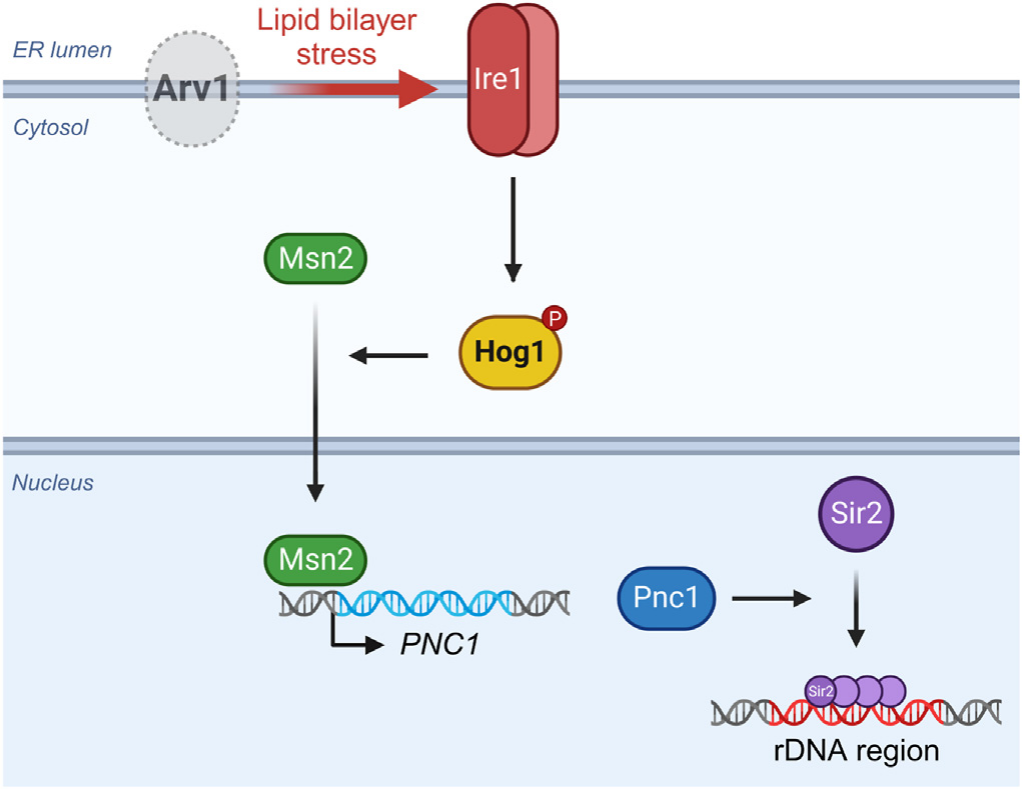
**A schematic summary of the mechanisms by which Arv1 deficiency increases rDNA stability.** The deletion of *ARV1* activates Ire1 and the UPR by inducing lipid bilayer stress. This activation leads to Ire1-mediated phosphorylation and activation of the MAP kinase Hog1. Subsequently, activated Hog1 enhances rDNA stability by inducing the nuclear localization of Msn2, the expression of Pnc1, and the association of Sir2 with rDNA. HOG, high osmolarity glycerol; MAP, mitogen-activated protein; rDNA, ribosomal DNA; UPR, unfolded protein response.

In this study, we elucidated the mechanism by which the loss of Arv1 increases rDNA stability, as summarized in Figure 6. The deletion of *ARV1* activates Ire1 and the UPR by inducing lipid bilayer stress. This activation leads to Ire1-mediated phosphorylation and activation of the MAP kinase Hog1. Subsequently, activated Hog1 enhances rDNA stability by inducing the nuclear localization of Msn2, the expression of Pnc1, and the association of Sir2 with rDNA. Meanwhile, *ARV1* deletion also activates the MAP kinase Slt2. While it is known that Slt2 activation can stimulate the nuclear translocation of Msn2 by downregulating PKA (47, 56), the downregulation of PKA or Slt2-dependent regulation of rDNA stability was not observed in *arv1*Δ cells (Figs. S1 and S5*A*). Typically, the downregulation of TORC1 and the activation of Snf1 can activate Msn2 (11). However, neither the downregulation of TORC1 nor the activation of Snf1 was observed in *arv1*Δ cells (Fig. S5). The Msn2/4-Pnc1-Sir2 pathway is a well-known rDNA stability regulatory pathway influenced by various upstream signaling pathways. Our findings suggest that in *arv1*Δ cells, the activation of the Msn2/4-Pnc1-Sir2 pathway is regulated through Hog1 but not by PKA, TORC1, or Snf1.

Considering that Sir2-mediated rDNA silencing inhibits the formation of ERCs, which are toxic to cells and contribute to replicative aging in *S. cerevisiae* (3, 4), it is plausible that the enhanced rDNA silencing and stability in *arv1*Δ cells could lead to an extension of the RLS. To investigate this possibility, we attempted to measure the RLS of *arv1*Δ cells. However, *arv1*Δ cells proved challenging for conventional micromanipulation due to severe aggregation (data not shown). A similar case has been reported previously. In *S. cerevisiae* cells lacking the *GAS1* gene, encoding a β-1,3-glucanosyltransferase, enhanced Sir2-mediated rDNA silencing is observed, but these cells cannot be separated by micromanipulation due to severe aggregation (47, 57). Since the loss of Arv1 induces the accumulation of immature Gas1 (54), Gas1 dysfunction resulting from *ARV1* deletion may lead to severe aggregation in *arv1*Δ cells. Further studies are needed to elucidate the aggregation phenotype of *S. cerevisiae* and to explore alternative methods for measuring the RLS in *arv1*Δ cells.

Both the protein *O*-mannosyltransferase Pmt1 and the GPI inositol deacylase Bst1 play roles in the ER homeostasis, and their dysfunction leads to the activation of the UPR (53, 58). The deletion of either *PMT1* or *BST1* is known to increase the RLS in an Ire1-dependent manner, but the detailed mechanism has not been elucidated (23, 24). It is presumable that an extension of the RLS in *pmt1*Δ and *bst1*Δ cells might be related to the enhancement of the protein quality control mechanism due to UPR activation. However, in this study, we discovered that rDNA silencing and stability increase in *pmt1*Δ and *bst1*Δ cells in a Hog1-dependent manner. Our findings provide insights into the contribution of rDNA stability to the molecular mechanisms by which Pmt1 and Bst1 regulate yeast lifespan.

As with Arv1 deficiency, rDNA stability increased in a Hog1-dependent manner under inositol depletion conditions, which induce lipid bilayer stress, and even under tunicamycin treatment conditions, which induce proteotoxic stress. Additionally, it is known that the deletion of *BST1* causes only proteotoxic stress, not lipid bilayer stress (38). This observation prompts the speculation that ER stress itself might have the capacity to enhance rDNA silencing and stability. However, it is important to note that not all instances of ER stress increase rDNA silencing. For instance, the impairment of Alg12, an α-1,6-mannosyltransferase localized in the ER, activates the UPR and extends the RLS in an Ire1-dependent manner, similar to the effects of Pmt1 or Bst1 dysfunction (23, 59, 60). However, unlike *pmt1*Δ and *bst1*Δ cells, rDNA silencing was not affected in *alg12*Δ cells (Fig. S6). It is likely that ER stress or UPR activation alone might be insufficient to regulate rDNA stability, and additional factors, other than rDNA stability, might contribute to the regulation of the RLS in *alg12*Δ cells.

Genes that share similar functions manifest similar profiles of genetic interactions (61). By comparing the similarity of the genetic interaction profiles of *ARV1*, *PMT1*, *BST1*, and *ALG12*, we found that *ARV1*, *BST1*, and *PMT1* show highly similar genetic interaction profiles, whereas the genetic interaction profile of *ALG12* displays relatively lower similarity to the other three genes (62). This observation suggests the possibility that certain attributes might be exclusively shared among *arv1*Δ, *pmt1*Δ, and *bst1*Δ cells, distinguishing them from *alg12*Δ cells, and potentially contributing to rDNA stability along with the UPR. Further investigations are warranted to uncover the specific factors responsible for the distinct regulation of rDNA stability in these mutants.

When the UPR is activated, overall protein synthesis slows down, and mechanisms for protein quality control, such as ER-associated degradation or autophagy, are initiated (16, 63, 64). Considering that protein homeostasis significantly contributes to the maintenance of the RLS along with rDNA stability (4, 23), the amplification of ER stress-induced protein homeostasis is expected to correlate with an extension of the RLS. Supporting this idea, the deletion of *RER1*, a gene involved in retrieving membrane proteins from the Golgi apparatus to the ER, extends the RLS in an autophagy-dependent manner (25). In this study, we demonstrated that ER stress can enhance rDNA stability, highlighting its dual contribution to protein homeostasis and rDNA stability—both major factors for RLS maintenance. Consequently, ER stress emerges as a pivotal player governing diverse regulatory mechanisms of the RLS, significantly influencing longevity.

The evolutionary conservation of Arv1, Ire1, Hog1, Msn2/4, Sir2, and the functional human ortholog of Pnc1 underscores their significance across the spectrum from yeast to humans (65). Notably, homologs of Hog1, namely p38 and JNK, are implicated in intricate signaling pathways, including the UPR, in mammalian systems (66). Furthermore, rDNA instability is linked to aging and diverse pathological conditions such as cancer in mammals (67, 68). Therefore, investigations into the regulatory mechanisms governing rDNA stability in yeast, as elucidated in this study, will provide valuable insights into understanding aging and disease pathogenesis in mammals.

## Experimental procedures

### Yeast strains, plasmids, and growth conditions

The yeast strains used in this study are listed in Table S1. Yeast cells were grown in YPD medium (1% yeast extract, 2% peptone, and 2% glucose), or synthetic complete (SC) medium (0.67% yeast nitrogen base without amino acids, 2% glucose and nutritional supplements) lacking appropriate amino acids, and all cultures were incubated at 30 °C. Tunicamycin was added to SC medium at a final concentration of 0.05 μg/ml and treated for 3 h. Inositol-depleted medium was prepared by combining pure chemicals based on the Difco & BBL Manual. Before switching to the inositol-depleted medium, cells were washed three times with triple distilled water. Gene disruption was performed using the one-step PCR-based gene targeting procedure (69). Yeast transformation was performed using the lithium acetate/single-stranded carrier DNA/PEG method (70), and confirmed by PCR.

The oligonucleotide primers used in this study are listed in Table S2. To express Ire1ΔIII, DNA fragments containing *pIRE1-IRE1-GFP* sequences were amplified by PCR and cloned into the NotI/KpnI-digested pRG203MX vector using the sequence- and ligation-independent cloning method (71, 72). Subsequently, mutations to generate Ire1ΔIII mutants were introduced by inverse PCR (73). *pIRE1* contains the sequence (∼1000 bp) upstream of the *IRE1* start codon. For reexpression of Arv1, DNA fragments containing *pARV1-ARV1-TAP* sequences were amplified by PCR and cloned into the NotI/KpnI-digested pRG203MX vector. *pARV1* contains the sequence (∼500 bp) upstream of the *ARV1* start codon. All vectors were digested with AscI and integrated into the *HIS3* locus.

### Analysis of HAC1 mRNA splicing

Total RNA was isolated from yeast cells using either the RNeasy Mini Kit (Qiagen) or the hot phenol:chloroform:isoamyl alcohol extraction with SDS method (74), and complementary DNA was generated using the ReverTra Ace qPCR RT Kit (TOYOBO). The primers used for the amplification of *HAC1* are shown in Table S2. Amplified PCR products were visualized by agarose gel electrophoresis. Densitometry determinations were performed using ImageJ software (https://imagej.net/ij/).

### Real-time quantitative reverse transcription-PCR

Total RNA was isolated from yeast cells using either the RNeasy Mini Kit (Qiagen) or the hot principal component analysis extraction with SDS method (74), and complementary DNA was generated using the ReverTra Ace qPCR RT Kit (TOYOBO). Real-time PCRs were performed with the QuantStudio 3 Real-Time PCR System (Applied Biosystems) and the 2X Real-Time PCR Kit with SFCgreen I (BioFACT). The primers for the real-time PCR are shown in Table S2. For the relative transcript level calculation, the transcript levels of the *FKS2*, *GPD1*, *mURA3*, and *KAR2* genes were normalized against *TAF10* gene by the 2^−ΔΔCt^ method (75).

### Immunoblot analysis

Using lysis buffer with protease inhibitors (0.01% NP-40, 50 mM Tris–HCl at pH 7.5, 150 mM NaCl, 1 mM EDTA, 1 μg/ml leupeptin, 1 μg/ml pepstatin, 1 mM benzamidine, and 1 mM phenylmethylsulfonyl fluoride) and phosphatase inhibitors (10 mM sodium fluoride, 10 mM β-glycerolphosphate, 10 mM sodium orthovanadate, and 10 mM sodium pyrophosphate), exponentially growing cells were lysed. Samples were bead-beaten with 0.5 mm glass beads five times for 1 min at 4 °C and incubated on ice for 1 min between bead beatings. Lysates were clarified by centrifugation at 13,000 rpm for 10 min at 4 °C. Bradford assay was used to measure the protein concentration. Combined with 6X SDS sample buffer, protein samples were boiled for 10 min at 75 °C. SDS-PAGE was performed with 8% separating gel. Immunoblot analysis was carried out by standard methods using a horseradish peroxidase (HRP)-conjugated mouse anti-GFP antibody (sc-9996, Santa Cruz Biotechnology) and an HRP-conjugated anti-mouse IgG antibody (A9044, Sigma-Aldrich). Phosphorylated Slt2 and Hog1 was detected by an anti-phospho-p44/42 MAPK (Erk1/2) (Thr202/Tyr204) antibody (#4370, Cell Signaling Technology) and anti-phospho-p38 MAPK (Thr180/Tyr182) antibody (#4511, Cell Signaling Technology), respectively. Act1, a loading control, was detected by HRP-conjugated mouse anti-actin antibody (sc-40, Santa Cruz Biotechnology). Images were captured using a luminescent image analyzer AE-9150 Ez-Capture II (ATTO) and CS analyzer version 3.0 software (ATTO, https://www.attoeng.site/cs-analyzer4). Densitometry determinations were performed using ImageJ software.

### Fluorescence microscopy

Yeast cells were grown in SC medium, and fluorescence microscopy was performed using a Nikon Eclipse E1 microscope with a Plan Fluor 100 × /1.30 numerical aperture oil immersion objective. Image analysis was conducted using NIS Elements imaging software (Nikon, https://www.microscope.healthcare.nikon.com/products/software/nis-elements). The percentage of cells with predominately nuclear fluorescence was determined by counting approximately 200 cells per strain.

### ChIP assay

ChIP assays were performed as previously described (5). Yeast cultures (100 ml) grown to an *A*_600_ of 1.0 were cross-linked with 1% formaldehyde for 15 min. Glycine was used to quench the reaction for 5 min at a final concentration of 125 mM. Cold phosphate-buffered saline was used for two rounds of cell washing. Cells were lysed by suspending cells in 400 μl of lysis buffer with protease inhibitors (0.1% SDS, 50 mM Hepes–KOH at pH 7.5, 1 mM EDTA at pH 8.0, 500 mM NaCl, 1% Triton X-100, 0.1% sodium deoxycholate, 1 mg/ml leupeptin, 1 mg/ml pepstatin, 1 mM benzamidine, and 1 mM PMSF). Samples were bead-beaten with 0.5 mm glass beads ten times for 1 min at 4 °C and incubated on ice for 2 min between bead beatings. Lysates were sonicated ten times for 15 s with amplitude set at 15% and incubated on cold water for 45 s between each sonication. Lysates were clarified by centrifugation at 13,000 rpm for 20 min at 4 °C. The DNA concentration was quantified and diluted to 1 mg/ml with lysis buffer. Subsequently, 100 μl of each lysate was used to obtain input DNA. For ChIP experiments, 100 μl of a 50% slurry of prewashed immunoglobulin G-agarose beads (Amersham Biosciences) was incubated with 1 ml of lysate at 4 °C overnight. Beads were washed twice in lysis buffer, once with wash buffer (10 mM Tris–HCl at pH 8.0, 1 mM EDTA at pH 8.0, 0.25 M LiCl, 0.5% NP-40, and 0.5% sodium deoxycholate), and once with Tris-EDTA (TE) buffer at room temperature. A total of 50 μl of elution buffer (50 mM Tris–HCl at pH 8.0, 1% SDS, and 10 mM EDTA) was added to elute the beads, and the beads with buffer were incubated at 65 °C for 10 min. Eluate was transferred to a fresh tube and gathered with a final bead wash of 150 μl of elution buffer. As to input DNA, 100 μl of elution buffer was added to 100 μl of lysate. Samples were incubated at 65 °C overnight, combined with 250 μg of proteinase K and 300 μl of TE, and incubated at 37 °C for 2 h. All samples were extracted once with phenol:chloroform:isoamyl alcohol and once with chloroform. 20 micrograms of glycogen and NaOAc (at a final concentration of 300 mM) was added to 400 μl of the sample. Precipitated DNA was resuspended in 100 μl of TE with 1 μg of RNase A and incubated at 37 °C for 2 h. ChIP samples were analyzed by quantitative real-time PCR using the QuantStudio 3 Real-Time PCR System (Applied Biosystems) and the 2X Real-Time PCR Kit with SFCgreen I (BioFACT). The primers used for PCR are shown in Table S2.

### rDNA silencing assay

Silencing at the rDNA region was tested as described previously (49, 76). Yeast cells were grown to an *A*_600_ of 1.0. Two microliters of 10-fold serial dilutions of the cell suspensions was spotted on SC and SC-Ura plates. Plates were incubated at 30 °C for 2 days before visualization.

### rDNA recombination assay

The frequency of the loss of *ADE2* integrated at the rDNA locus of strain DMY3010 was measured to determine the rDNA recombination rate as described previously (7). Yeast cells were grown to an *A*_600_ of 1.0 and spread on SC plates. Colonies were grown for 2 days at 30 °C and placed at 4 °C for 2 days to turn red. The rDNA recombination rate was estimated by dividing the number of half-red/half-white colonies by the total number of colonies. Totally red colonies were excluded. Approximately 10,000 cells per strain were counted to determine the rDNA recombination rate.

### Statistical analysis

Values represent the average of three independent experiments, and error bars indicate the standard deviation. Statistical analysis was performed by a paired two-tailed Student’s *t* test: ∗*p* < 0.05; ∗∗*p* < 0.01; ∗∗∗*p* < 0.001; ns, not significant.

## Data availability

All data are contained within the article.

## Supporting information

This article contains supporting information (47, 49, 77, 78, 79, 80).

## Conflict of interest

The authors declare that they have no conflicts of interest with the contents of this article.
