# Supplementary material for: ARV1 deficiency induces lipid bilayer stress and enhances rDNA stability by activating the unfolded protein response in Saccharomyces cerevisiae
Source: J Biol Chem. 2024 Apr 6;300(5):107273. doi: 10.1016/j.jbc.2024.107273 (PMC11089378; doi:10.1016/j.jbc.2024.107273)
Supplement: Supporting Information [file mmc1.pdf]

Supporting Information

ARV1 deficiency induces lipid bilayer stress and enhances rDNA stability by activating the unfolded protein response in *Saccharomyces cerevisiae*

Sujin Hong[1,‡], Hyeon-geun Lee[1,‡], and Won-Ki Huh[1,2]*

[1]School of Biological Sciences, Seoul National University, Seoul 08826, Republic of Korea.
[2]Institute of Microbiology, Seoul National University, Seoul 08826, Republic of Korea

‡ These authors contributed equally to this work.
* For correspondence: Won-Ki Huh, wkh@snu.ac.kr.

Table of Contents

Experimental Procedures

Figure S1. Loss of Arv1 enhances Sir2-mediated rDNA silencing and rDNA stability in a Slt2-independent manner.
Figure S2. Various ER stresses enhance rDNA silencing.
Figure S3. Various ER stresses enhance rDNA stability.
Figure S4. Overexpression of Arv1 does not induce UPR and does not increase rDNA stability
Figure S5. Loss of Arv1 does not inhibit PKA or TORC1.
Figure S6. Loss of Alg12 does not affect rDNA silencing and rDNA stability.
Table S1. Yeast strains used in this study.
Table S2. Oligonucleotide primers used in this study.

Experimental Procedures

Measurement of Cki1 phosphorylation

Total protein extraction and western blot analysis was performed as described previously (1). Myc-tagged Cki1 proteins are detected using a HRP-conjugated mouse anti-Myc antibody (sc-40 HRP, Santa Cruz Biotechnology). Images were captured Using a luminescent image analyzer AE-9150 Ez-Capture II (ATTO) and CS analyzer version 3.0 software (ATTO). Densitometry determinations was performed using ImageJ software.

Measurement of Sch9 phosphorylation

Analysis of phosphorylated Sch9 was conducted by western blotting as described previously (2). Cells were grown to log phase and trichloroacetic acid was added up to 6%. Samples were put on ice for at least 5 min, spun down, washed twice with cold acetone, and dried. Cells were bead-beaten in 100 μl of urea buffer (6Murea, 50 mM Tris-HCl, pH 7.5, 5 mM EDTA, 1% SDS, 1 mM phenylmethylsulfonyl fluoride, 5 mM NaF, 5mM NaN3, 5mM p-nitrophenyl phosphate, 5 mM Na2P2O4, and 5 mM β-glycerophosphate) followed by heating for 10 min to 65°C. For 2-nitro-5-thiocyanobenzoic acid cleavage, 30 μl of 0.5M CHES (pH 10.5) and 20 μl of 2-nitro-5-thiocyanobenzoic acid (7.5 mM in H2O) were added, and samples were incubated overnight at room temperature before adding 6X SDS sample buffer. Sch9 phosphorylation was detected by SDS-PAGE and immunoblotting using HRP-conjugated mouse anti-HA antibody (sc-7392 HRP, Santa Cruz Biotechnology). Images were captured Using a luminescent image analyzer AE-9150 Ez-Capture II (ATTO) and CS analyzer version 3.0 software (ATTO). Densitometry determinations was performed using ImageJ software.

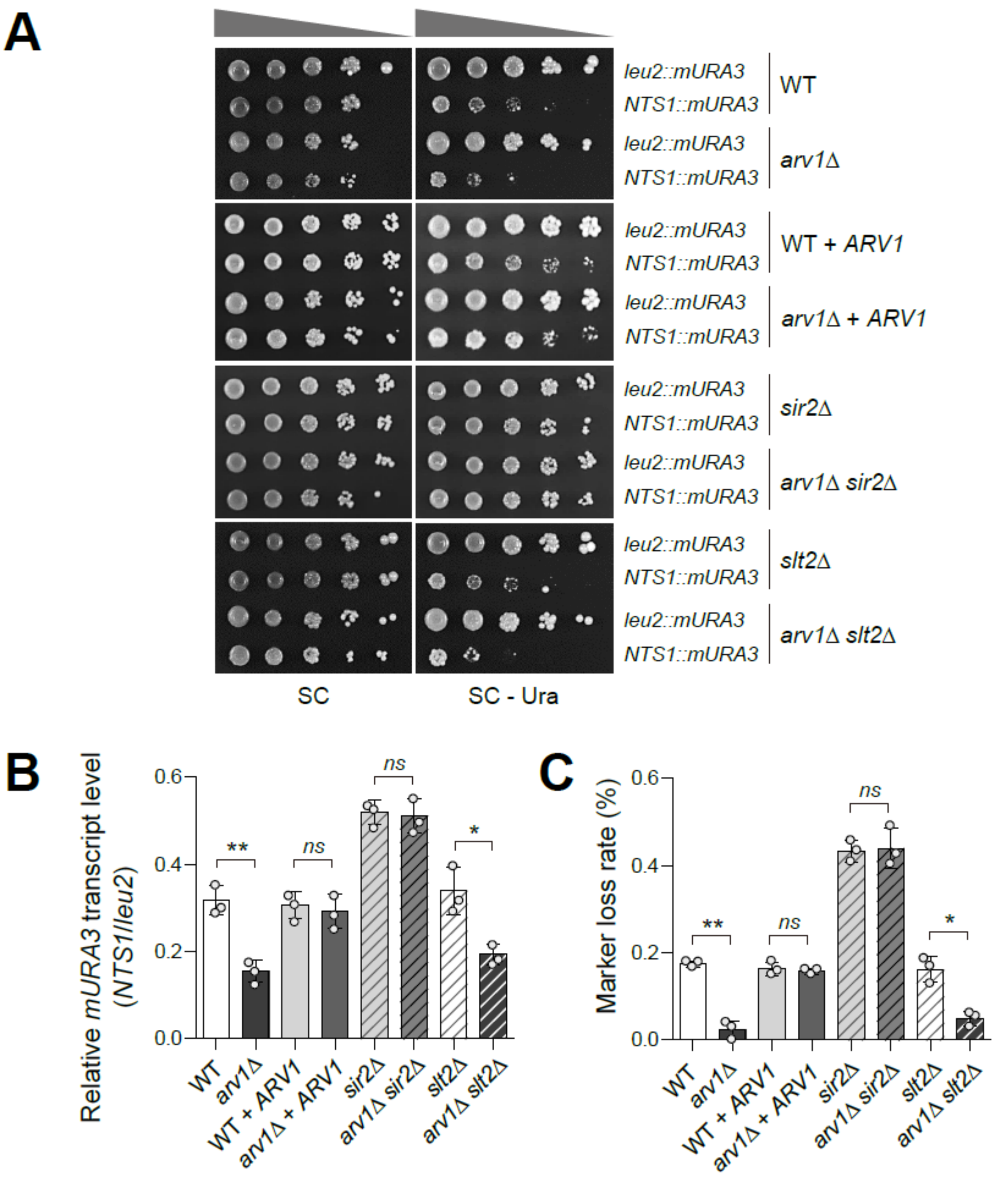

Figure S1. Loss of Arv1 enhances Sir2-mediated rDNA silencing and rDNA stability in a Slt2-independent manner. *A,* rDNA silencing assay performed with indicated cells. WT + *ARV1 and arv1*Δ + *ARV1* indicate wild-type (WT) and *arv1*Δ cells expressing *ARV1-TAP* under its native promoter. Silencing at the rDNA region was assessed by monitoring the growth of 10-fold serial dilutions of cells on SC media lacking uracil. SC medium was used as a plating control. *B,* Relative *mURA3* transcript levels in the indicated cells. Total RNA was extracted and analyzed by quantitative real-time reverse transcription-PCR. The relative transcript levels of the *mURA3* gene were calculated as the ratio of the normalized transcript levels of the *mURA3* gene inside the rDNA array (*NTS1::mURA3*) to those outside the rDNA array (*leu2::mURA3*). *C,* rDNA recombination assay performed with the indicated cells. rDNA recombination is represented by the frequency of loss of the *ADE2* marker gene integrated at the rDNA locus in the corresponding cells. For *B* and *C*, values represent the average of three independent experiments, and error bars indicate the standard deviation. Asterisks indicate significant differences (paired two-tailed Student's *t*-test): *$P < 0.05$; **$P < 0.01$; ns, not significant.

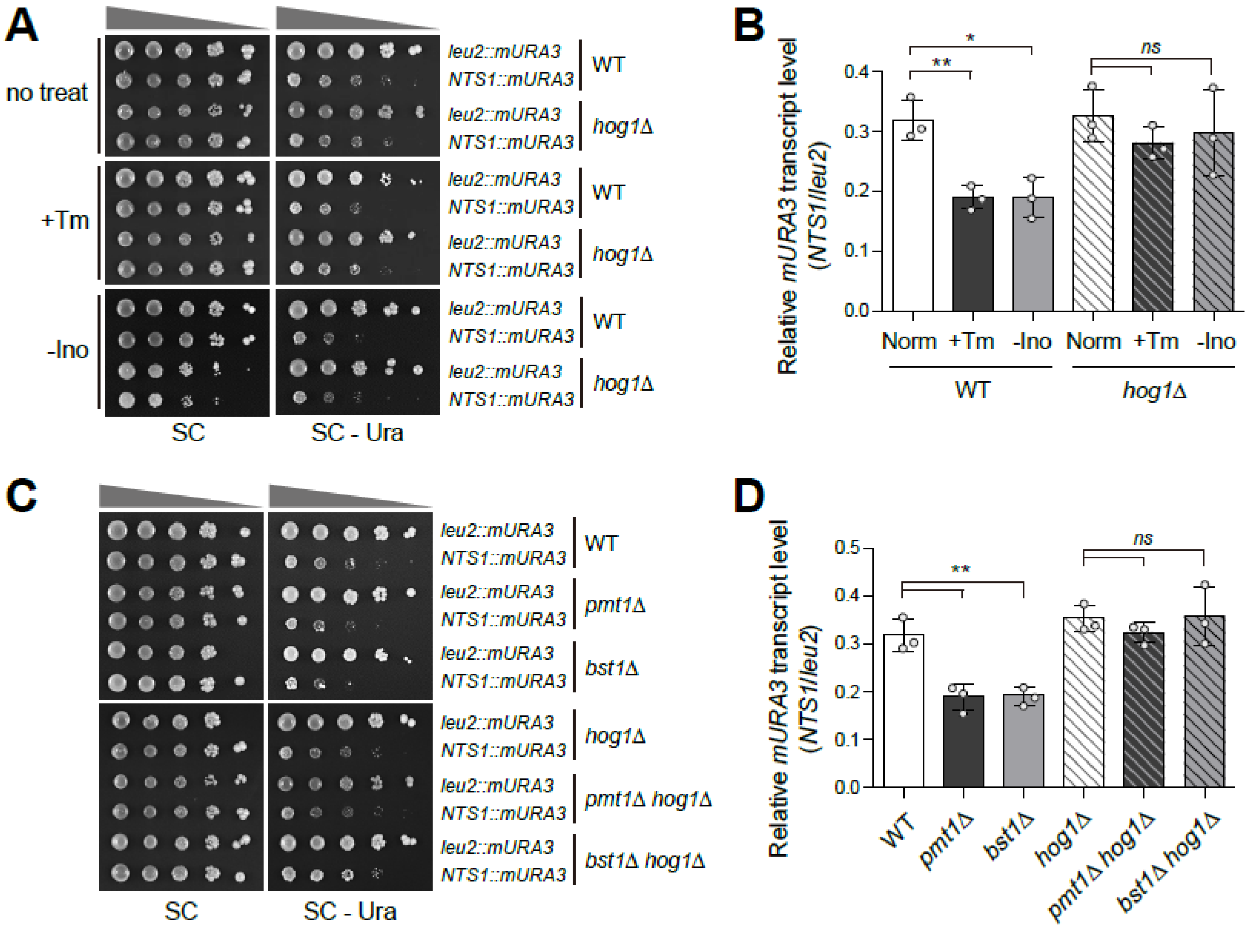

Figure S2. Various ER stresses enhance rDNA silencing. *A,* rDNA silencing assay performed with wild-type (WT) and *hog1*Δ cells the indicated cells under 0.05 μg/ml Tm treatment or inositol depletion. *B,* Relative *mURA3* transcript levels in the indicated cells. *C,* rDNA silencing assay performed with WT, *pmt1*Δ, *bst1*Δ, *hog1*Δ, *pmt1*Δ *hog1*Δ, and *bst1*Δ *hog1*Δ cells. *D,* Relative *mURA3* transcript levels in the indicated cells. For *A* and *C*, silencing at the rDNA region was assessed by monitoring the growth of 10-fold serial dilutions of cells on SC media lacking uracil. SC medium was used as a plating control. For *B* and *D*, Total RNA was extracted and analyzed by quantitative real-time reverse transcription-PCR. The relative transcript levels of the *mURA3* gene were calculated as the ratio of the normalized transcript levels of the *mURA3* gene inside the rDNA array (*NTS1::mURA3*) to those outside the rDNA array (*leu2::mURA3*). Values represent the average of three independent experiments, and error bars indicate the standard deviation. Asterisks indicate significant differences (paired two-tailed Student's *t*-test): $^*P < 0.05$; $^{**}P < 0.01$; ns, not significant.

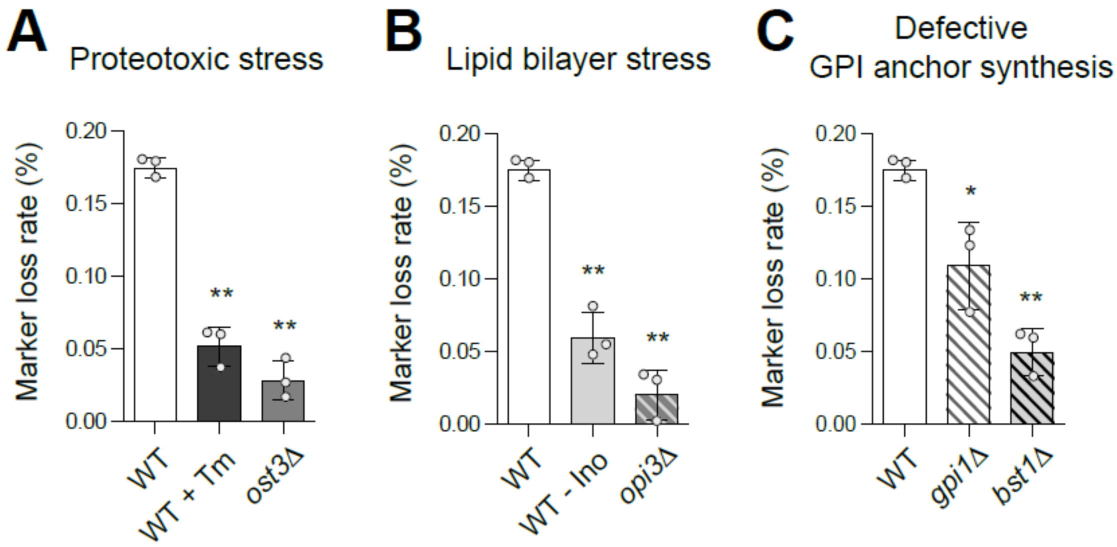

Figure S3. Various ER stresses enhance rDNA stability. The rDNA recombination assay was performed with the indicated cells treated with or without 0.05 μg/ml tunicamycin (Tm) or inositol for 3 h. rDNA recombination is represented by the frequency of loss of the *ADE2* marker gene integrated at the rDNA locus in the corresponding cells. Values represent the average of three independent experiments, and error bars indicate the standard deviation. Asterisks indicate significant differences (paired two-tailed Student's *t*-test): *$P < 0.05$; **$P < 0.01$; ns, not significant.

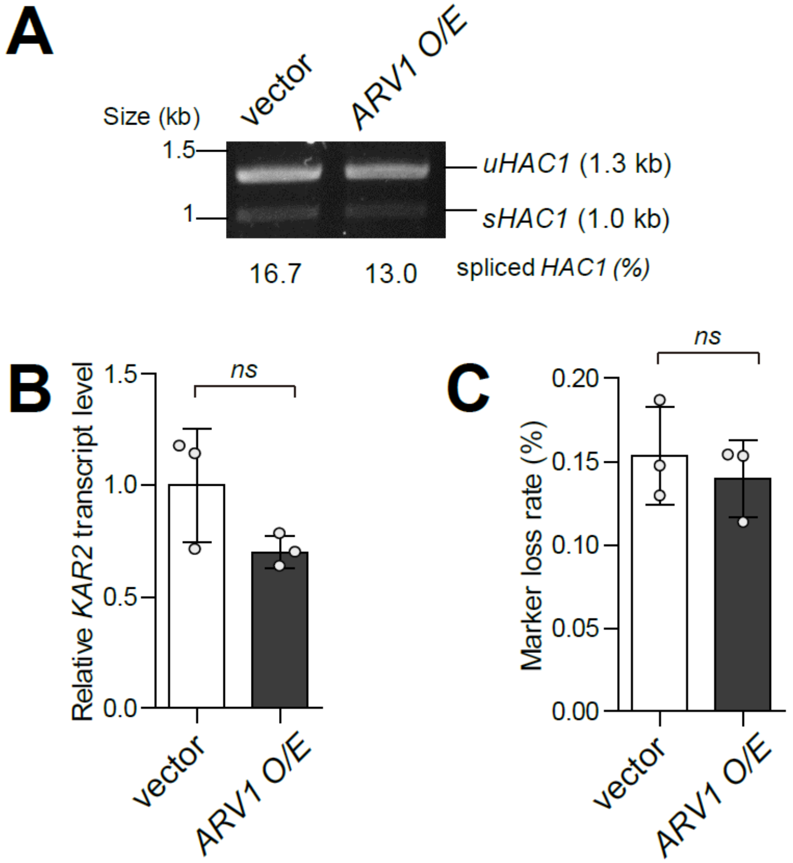

Figure S4. Overexpression of Arv1 does not induce UPR and does not increase rDNA stability. *A*, Analysis of *HAC1* mRNA splicing in wild-type cells harboring *p413ADH or p413ADH-ARV1-TAP* vector. *B,* Relative *KAR2* transcript levels in the indicated cells Total RNA was extracted and analyzed by quantitative real-time reverse transcription-PCR. The relative *KAR2* transcript level was normalized against *TAF10* and calculated using the $2^{-\Delta\Delta Ct}$ method. *C*, rDNA recombination assay performed with the indicated cells. rDNA recombination is represented by the frequency of loss of the *ADE2* marker gene integrated at the rDNA locus in the corresponding cells. For *C* and *D*, values represent the average of three independent experiments, and error bars indicate the standard deviation.

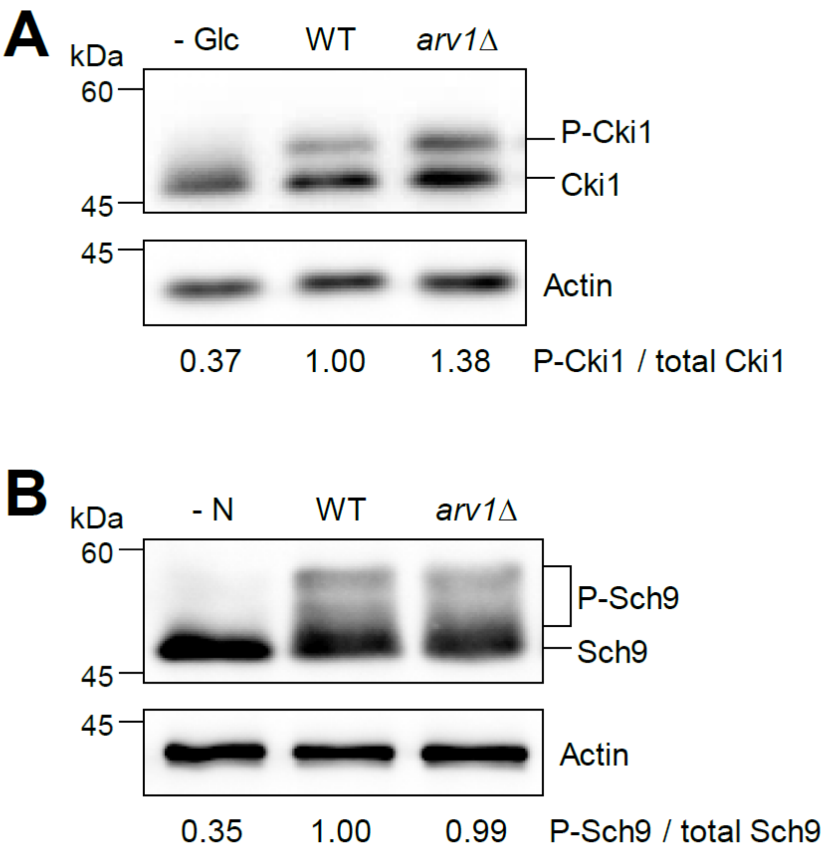

Figure S5. Loss of Arv1 does not inhibit PKA or TORC1. *A*, Activity of PKA was examined by monitoring phosphorylation of Cki1. *B*, Activity of TORC1 was examined by monitoring phosphorylation of Sch9. In wild-type (WT) and *arv1*Δ cells, total protein was extracted and immunoblotting was performed using a mouse anti-Myc antibody for detection of Myc-tagged Cki1 or a mouse anti-HA antibody for detection of HA-tagged Sch9. Act1 was used as a loading control. The relative ratio of phosphorylated protein to total protein, normalized against that of WT cells, is shown below each lane.

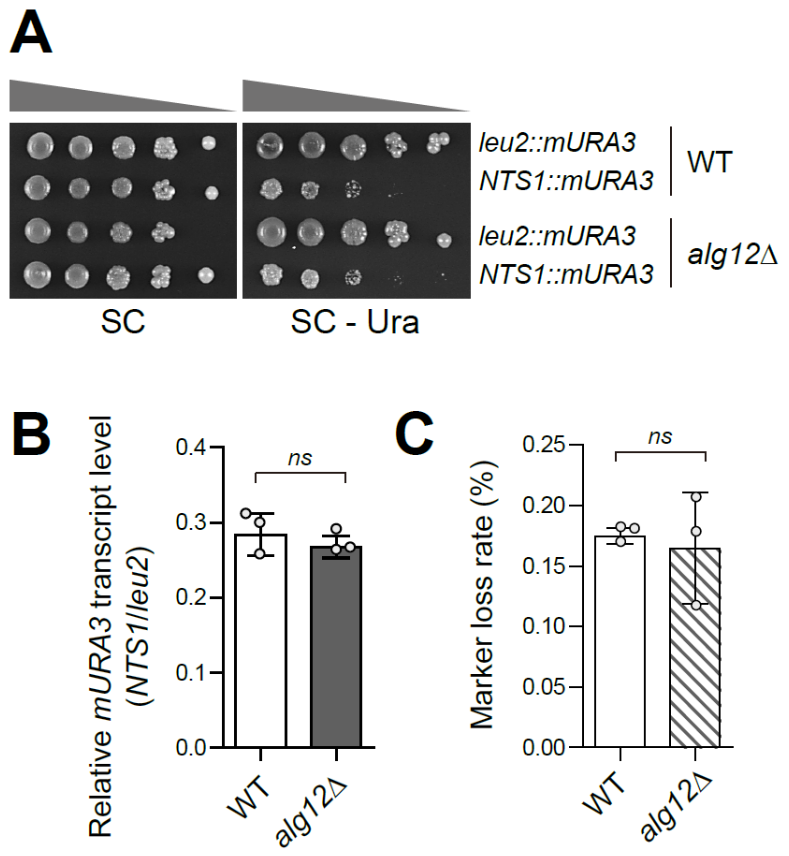

Figure S6. Loss of Alg12 does not affect rDNA silencing. *A,* rDNA silencing assay performed with wild-type (WT) and *alg12*Δ cells. Silencing at the rDNA region was assessed by monitoring the growth of 10-fold serial dilutions of cells on SC media lacking uracil. SC medium was used as a plating control. *B,* Relative *mURA3* transcript levels in WT and *alg12*Δ cells. Total RNA was extracted and analyzed by quantitative real-time reverse transcription-PCR. The relative transcript levels of the *mURA3* gene were calculated as the ratio of the normalized transcript levels of the *mURA3* gene inside the rDNA array (*NTS1::mURA3*) to those outside the rDNA array (*leu2::mURA3*). *C*, rDNA recombination assay performed with WT and *alg12*Δ cells. rDNA recombination is represented by the frequency of loss of the *ADE2* marker gene integrated at the rDNA locus in the corresponding cells. Values represent the average of three independent experiments, and error bars indicate the standard deviation. Statistical analysis was performed using a two-tailed Student's *t*-test. ns, not significant.

Table S1. Yeast strains used in this study.

Strain	Genotype	Source
BY4741	*MAT*a *his3Δ1 leu2Δ0 met15Δ0 ura3Δ0*	EUROSCARF
HY2543	BY4741; *arv1Δ::LEU2*	This study
HY2710	BY4741; *ire1Δ::URA3 pRG203-IRE1-GFP*	This study
HY2711	BY4741; *ire1Δ::URA3 pRG203-IRE1ΔIII-GFP*	This study
HY2712	BY4741; *ire1Δ::URA3 arv1Δ::KanMX4 pRG203-IRE1-GFP*	This study
HY2713	BY4741; *ire1Δ::URA3 arv1Δ::KanMX4 pRG203-IRE1ΔIII-GFP*	This study
HY2689	BY4741; *SLT2-GFP-HIS3MX6*	(3)
HY2690	HY2689; *arv1Δ::URA3*	This study
HY2691	HY2689; *ire1Δ::LEU2*	This study
HY2692	HY2689; *arv1Δ::URA3 ire1Δ::LEU2*	This study
HY2694	BY4741; *slt2Δ::LEU2*	This study
HY2695	BY4741; *slt2Δ::LEU2 arv1Δ::KanMX4*	This study
HY2209	BY4741; *HOG1-GFP-HIS3MX6*	(3)
HY2527	HY2209; *arv1Δ::LEU2*	This study
HY2528	HY2209; *ire1Δ::URA3*	This study
HY2529	HY2209; *arv1Δ::LEU2 ire1Δ::URA3*	This study
DMY2798	*MAT*a *ade2-1 ura3-1 trp1-1 leu2-3,112 his3-11 can1-100 leu2::mURA3*	(4)
DMY2804	*MAT*a *ade2-1 ura3-1 trp1-1 leu2-3,112 his3-11 can1-100 RDN1-NTS1::mURA3*	(4)
HY2545	DMY2798; *arv1Δ::KanMX4*	This study
HY2556	DMY2804; *arv1Δ::KanMX4*	This study

HY2546	DMY2798; *arv1Δ::KanMX4 pRG203-ARV1-TAP*	This study
HY2557	DMY2804; *arv1Δ::KanMX4 pRG203-ARV1-TAP*	This study
HY2547	DMY2798; *sir2Δ::TRP1*	This study
HY2558	DMY2804; *sir2Δ::TRP1*	This study
HY2548	DMY2798; *sir2Δ::TRP1 arv1Δ::HIS3*	This study
HY2559	DMY2804; *sir2Δ::TRP1 arv1Δ::HIS3*	This study
HY2696	DMY2798; *slt2Δ::HIS3*	This study
HY2697	DMY2804; *slt2Δ::HIS3*	This study
HY2698	DMY2798; *slt2Δ::HIS3 arv1Δ::KanMX4*	This study
HY2699	DMY2804; *slt2Δ::HIS3 arv1Δ::KanMX4*	This study
HY2549	DMY2798; *hog1Δ::HIS3*	This study
HY2560	DMY2804; *hog1Δ::HIS3*	This study
HY2550	DMY2798; *hog1Δ::HIS3 arv1Δ::TRP1*	This study
HY2561	DMY2804; *hog1Δ::HIS3 arv1Δ::TRP1*	This study
HY2551	DMY2798; *ire1Δ::HIS3*	This study
HY2562	DMY2804; *ire1Δ::HIS3*	This study
HY2552	DMY2798; *ire1Δ::HIS3 arv1Δ::TRP1*	This study
HY2563	DMY2804; *ire1Δ::HIS3 arv1Δ::TRP1*	This study
DMY3010	*MAT*a *ade2-1 ura3-1 trp1-1 leu2-3,112 his3-11 can1-100 RAD5*$^{+}$ with *RDN1::ADE2*	(4)
HY2567	DMY3010; *arv1Δ::TRP1*	This study
HY2568	DMY3010; *arv1Δ::TRP1 pRG203-ARV1-TAP*	This study
HY2569	DMY3010; *sir2Δ::HIS3*	This study

HY2570	DMY3010; *sir2Δ::HIS3 arv1Δ::TRP1*	This study
HY2700	DMY3010; *slt2Δ::LEU2*	This study
HY2701	DMY3010; *slt2Δ::LEU2 arv1Δ::TRP1*	This study
HY2571	DMY3010; *hog1Δ::HIS3*	This study
HY2572	DMY3010; *hog1Δ::HIS3 arv1Δ::TRP1*	This study
HY2573	DMY3010; *ire1Δ::HIS3*	This study
HY2574	DMY3010; *ire1Δ::HIS3 arv1Δ::TRP1*	This study
HY1170	BY4741; *MSN2-GFP-HIS3MX6*	(3)
HY2515	HY1170; *arv1Δ::LEU2*	This study
HY2516	HY1170; *hog1Δ::URA3*	This study
HY2517	HY1170; *arv1Δ::LEU2 hog1Δ::URA3*	This study
HY1172	BY4741; *MSN2-TAP-HIS3MX6*	(5)
HY2521	HY1172; *arv1Δ::LEU2*	This study
HY2522	HY1172; *hog1Δ::URA3*	This study
HY2523	HY1172; *arv1Δ::LEU2 hog1Δ::URA3*	This study
HY1174	BY4741; *PNC1-GFP-HIS3MX6*	(3)
HY2535	HY1174; *arv1Δ::LEU2*	This study
HY1176	HY1174; *msn2Δ::KanMX4 msn4Δ::LEU2*	(6)
HY2533	HY1176; *arv1Δ::URA3*	This study
HY2538	HY1174; *hog1Δ::URA3*	This study
HY2539	HY1174; *arv1Δ::LEU2 hog1Δ::URA3*	This study
HY1178	BY4741; *SIR2-TAP-HIS3MX6*	(5)

HY2531	HY1178; *arv1Δ::URA3*	This study
HY1180	HY1178; *msn2Δ::KanMX4 msn4Δ::LEU2*	(6)
HY2533	HY1180; *arv1Δ::URA3*	This study
HY2583	DMY2798; *pmt1Δ::TRP1*	This study
HY2585	DMY2804; *pmt1Δ::TRP1*	This study
HY2584	DMY2798; *pmt1Δ::TRP1 hog1Δ::HIS3*	This study
HY2586	DMY2804; *pmt1Δ::TRP1 hog1Δ::HIS3*	This study
HY2681	DMY2798; *bst1Δ::HIS3*	This study
HY2682	DMY2804; *bst1Δ::HIS3*	This study
HY2683	DMY2798; *bst1Δ::HIS3 hog1Δ::TRP1*	This study
HY2684	DMY2804; *bst1Δ::HIS3 hog1Δ::TRP1*	This study
HY2587	DMY3010; *pmt1Δ::TRP1*	This study
HY2588	DMY3010; *pmt1Δ::TRP1 hog1Δ::HIS3*	This study
HY2687	DMY3010; *bst1Δ::HIS3*	This study
HY2688	DMY3010; *bst1Δ::HIS3 hog1Δ::TRP1*	This study
HY2702	BY4741; *p413ADH*	This study
HY2703	BY4741; *p413ADH-ARV1-TAP*	This study
HY2704	DMY3010; *p413ADH*	This study
HY2705	DMY3010; *p413ADH-ARV1-TAP*	This study
HY2706	BY4741; *pRS423-prCUP-6xMYC-cki12-200(S125/130A)*	This study
HY2707	BY4741; *arv1Δ::KanMX4 pRS423-prCUP-6xMYC-cki12-200(S125/130A)*	This study
HY2708	BY4741; *pRS416-SCH9(T570A)-5HA*	This study

HY2709	BY4741; *arv1Δ::KanMX4 pRS416-SCH9(T570A)-5HA*	This study
HY2685	DMY2798; *alg12Δ::HIS3*	This study
HY2686	DMY2804; *alg12Δ::HIS3*	This study
HY2687	DMY3010; *ost3Δ::URA3*	This study
HY2688	DMY3010; *opi3Δ::URA3*	This study
HY2689	DMY3010; *gpi1Δ::URA3*	This study

Table S2. Oligonucleotide primers used in this study.

Target	Forward Primer	Reverse Primer
**For plasmid construction**		
pRG203MX-IRE1-GFP	ATATTTCTTTTCGCGGCCGCGTCTTATCCTTGCCATAAAC	CGAAGCTGGGTACCGGGCCCTGCGCGCCCTATTTGTATAGTTCATCC
IRE1ΔIII mutagenesis	ACCAGAAGAACCTTTTGAACCAGGTCCGAACG	GGTTCTTCTGGTGAATGTGAAAATATGATTGTAATAGGC
pRG203MX-ARV1-TAP	ATATTTCTTTTCGCGGCCGCTATTAGCGCACCATTGGGGC	TAAATACGGCCGAAGCTGGGTACCGTCTCACTGATGATTCG
p413ADH-ARV1-TAP	ATGCTCTAGAATGATTTGCATAACGTGCATGC	ATGCCTCGAGTCACTGATGATTCGCGTCT
**For *HAC1* mRNA splicing analysis**		
HAC1 mRNA	GTCAAACATAACAACCTCCTC	TCAAGAGCTATGTTCAGTGTCG
**For real-time quantitative reverse transcription-PCR**		
TAF10	ATTCCAGGATCAGGTCTTCCGTAGC	TCTCATTCTGTTGATGTTGTTGTTG
FKS2	AAAAGACCTTGGATCTGGG	TAAGGATGGCGTATGAGTG
GPD1	GAAAAGGAGTTGTTGATGG	GCAGGTTCTTCATTGGGTAG
mURA3	CTGTTGACATTGCGAAGAGC	TCTCCCTTGTCATCTAAACC
KAR2	TCGAAGTCCAAGCCACTTCT	GCACGTTTAGCCTTTTCAGC
**For ChIP assay**		
PNC1 promoter	GATCAAGGTGGCACACAGGG	ATACATAGTGGGCCAAACGG
rDNA-25S	CGACTAACCCACGTCCAACT	CCGAATGAACTAGCCCTGAA
rDNA-NTS1	TCCCCACTGTTCACTGTTCA	AGGGCTTTCACAAAGCTTCC
rDNA-NTS2/18S	AAGATGCCCACGATGAGACT	GGGAGGTACTTCATGCGAAA
rDNA-18S	CCAGAACGTCTAAGGGCATC	CTCACCAGGTCCAGACACAA

References

1. Yi, D. G., Hong, S., and Huh, W. K. (2018) Mitochondrial dysfunction reduces yeast replicative lifespan by elevating RAS-dependent ROS production by the ER-localized NADPH oxidase Yno1. *PLoS One* **13**, e0198619
2. Urban, J., Soulard, A., Huber, A., Lippman, S., Mukhopadhyay, D., Deloche, O., Wanke, V., Anrather, D., Ammerer, G., Riezman, H., Broach, J. R., De Virgilio, C., Hall, M. N., and Loewith, R. (2007) Sch9 is a major target of TORC1 in. *Molecular Cell* **26**, 663-674
3. Huh, W.-K., Falvo, J. V., Gerke, L. C., Carroll, A. S., Howson, R. W., Weissman, J. S., and O'Shea, E. K. (2003) Global analysis of protein localization in budding yeast. *Nature* **425**, 686-691
4. Huang, J., Brito, I. L., Villén, J., Gygi, S. P., Amon, A., and Moazed, D. (2006) Inhibition of homologous recombination by a cohesin-associated clamp complex recruited to the rDNA recombination enhancer. *Genes & Development* **20**, 2887-2901
5. Ghaemmaghami, S., Huh, W.-K., Bower, K., Howson, R. W., Belle, A., Dephoure, N., O'Shea, E. K., and Weissman, J. S. (2003) Global analysis of protein expression in yeast. *Nature* **425**, 737-741
6. Ha, C. W., Kim, K., Chang, Y. J., Kim, B., and Huh, W.-K. (2014) The β-1, 3-glucanosyltransferase Gas1 regulates Sir2-mediated rDNA stability in Saccharomyces cerevisiae. *Nucleic Acids Research* **42**, 8486-8499
